# Pharmacological interventions for improving cannabis use and psychosis in dual diagnosis: a systematic review

**DOI:** 10.1192/bjo.2021.771

**Published:** 2021-06-18

**Authors:** Arundeep Singh Bharj, Katy Jones, Musa Sami

**Affiliations:** University of Nottingham

## Abstract

**Aims:**

Many patients with psychosis symptoms and schizophrenia use cannabis as a recreational drug. Patients who use cannabis respond differently to antipsychotic treatment compared to those who do not. Despite this, there is a lack of evidence, and therefore clinical guidance, pertaining to the best pharmacological treatment to improve psychosis or cannabis use in this population. This systematic review was carried out to assess the current evidence base regarding the most effective pharmacological treatment for patients with psychosis who also have a background of using cannabis. Our specific question was: ‘in patients with a dual diagnosis of psychosis and cannabis use, which pharmacological interventions have the most efficacy in improving psychosis or reducing cannabis use?’.

**Method:**

A search of EMBASE, PsychINFO, and MEDLINE(R) databases was carried out on September 30, 2020. Bibliographies of other studies were also searched for relevant articles. After exclusion of any articles which did not meet inclusion criteria for this review, eleven full texts remained; a qualitative analysis was carried out on these, but there was no meta-analysis. Only randomised control trials (RCTs) whose interventions and controls were pharmacological therapies, and which included patients with a background of cannabis use and psychosis, and which measured clinical outcomes, were included.

**Result:**

We found 11 articles which analysed 10 RCT studies (n = 363) investigating risperidone, olanzapine, clozapine, haloperidol, ziprasidone and imipramine. 6/11 were double blind. The studies were small in size, varied in their methodology, exact inclusion criteria, exact outcomes, and all had a high risk of bias. Few significant findings were found. There is limited evidence for clozapine having anti-craving effect however whether this is associated with reduction in use remains to be demonstrated. We found no studies of adjunctive anticonvulsant agents, which are often used in psychotic disorders.

**Conclusion:**

This review underlines the paucity of studies on which to make evidence-based decisions. No new studies have been undertaken since the last systematic review in this area in the last 7 years. Due to the lack of high-quality evidence found by this review, there remains a considerable need for interventional, high-quality RCTs in this comorbid patient group.

